# Comprehensive analysis of MHC class I genes from the U-, S-, and Z-lineages in Atlantic salmon

**DOI:** 10.1186/1471-2164-11-154

**Published:** 2010-03-05

**Authors:** Morten F Lukacs, Håvard Harstad, Hege G Bakke, Marianne Beetz-Sargent, Linda McKinnel, Krzysztof P Lubieniecki, Ben F Koop, Unni Grimholt

**Affiliations:** 1 Department of Basic Science and Aquatic Medicine, Norwegian School of Veterinary Science, Oslo, Norway; 2 Centre for Ecology and Evolutionary Synthesis, Dept of Biology, University of Oslo, Norway; 3 Department of Biology, University of Victoria, Victoria BC V8W 2Y2, Canada; 4 Department of Molecular Biology & Biochemistry, Simon Fraser University, Burnaby BC, Canada

## Abstract

**Background:**

We have previously sequenced more than 500 kb of the duplicated MHC class I regions in Atlantic salmon. In the IA region we identified the loci for the MHC class I gene *Sasa-UBA *in addition to a soluble MHC class I molecule, *Sasa-ULA*. A pseudolocus for *Sasa-UCA *was identified in the nonclassical IB region. Both regions contained genes for antigen presentation, as wells as orthologues to other genes residing in the human MHC region.

**Results:**

The genomic localisation of two MHC class I lineages (Z and S) has been resolved. 7 BACs were sequenced using a combination of standard Sanger and 454 sequencing. The new sequence data extended the IA region with 150 kb identifying the location of one Z-lineage locus, *ZAA*. The IB region was extended with 350 kb including three new Z-lineage loci, *ZBA*, *ZCA *and *ZDA *in addition to a *UGA *locus. An allelic version of the IB region contained a functional *UDA *locus in addition to the *UCA *pseudolocus. Additionally a BAC harbouring two MHC class I genes (UHA) was placed on linkage group 14, while a BAC containing the S-lineage locus *SAA *(previously known as *UAA*) was placed on LG10. Gene expression studies showed limited expression range for all class I genes with exception of *UBA *being dominantly expressed in gut, spleen and gills, and *ZAA *with high expression in blood.

**Conclusion:**

Here we describe the genomic organization of MHC class I loci from the U-, Z-, and S-lineages in Atlantic salmon. Nine of the described class I genes are located in the extension of the duplicated IA and IB regions, while three class I genes are found on two separate linkage groups. The gene organization of the two regions indicates that the IB region is evolving at a different pace than the IA region. Expression profiling, polymorphic content, peptide binding properties and phylogenetic relationship show that Atlantic salmon has only one MHC class Ia gene (*UBA*), in addition to a multitude of nonclassical MHC class I genes from the U-, S- and Z-lineages.

## Background

Major histocompatibility complex (MHC) class I molecules have important roles in presenting antigens to immune cells thereby enabling the organism to discriminate between self and non-self. In humans, the MHC genomic region is a gene dense region covering more than 4 Mb and encodes MHC class I and II molecules in addition to numerous other immune related molecules. The classical MHC region is located on chromosome 6, while duplicated regions are found on chromosomes 1, 9 and 19, resulting from two whole-genome duplications [1]. The primary form of the MHC class I molecule consists of an alpha chain, stabilized by a β2-microglobulin (β2 m) molecule.

MHC class I molecules are at present divided into two main categories; classical MHC class I (or Ia) and nonclassical MHC class I (or Ib) where the latter category contains a multitude of molecules with various ligand binding abilities. MHC class Ia molecules are extremely polymorphic and expressed in most cells where they present self and nonself peptides primarily to CD8+ T-cells. In humans the MHC class Ia molecules are encoded by the HLA-A, HLA-B and HLA-C loci. The MHC class Ib molecules are less polymorphic and have a more restricted tissue distribution. The MHC class Ib family include the peptide binders like HLA-E, HLA-F, HLA-G. Additionally lipid binders like CD1, non-ligand binders like MIC-A/B and HFE, and other molecules like MR1, ZAG, FcRn and ULBP have features of MHC class Ib character. An emerging view on the MHC class Ib molecules are their prominent role bridging the innate and acquired immunity [2].

Teleost fishes also display a wide variety of MHC class I molecules, and earlier reports classify these genes into the U-, Z- and L-lineage based on evolutionary relationship [3,4]. However information regarding polymorphic content, expression patterns and ligand binding are mostly lacking for these MHC class I genes. The broad U-lineage is present in most teleosts, covering both MHC class Ia and Ib sequences. Z-lineage molecules have now been identified in a multitude of teleosts [4-6]. L-lineage molecules are so far only found in salmonids and cyprinids, and represent highly divergent class Ib genes [3].

All teleost fishes have their MHC class I and II regions located on different linkage groups [7,8], an event thought to have emerged by a genome duplication event early in the teleost evolution [9]. A unique salmonid specific genome duplication event occurring 60 mya [10,11] resulted in a duplicated MHC class I region residing on different chromosomes in salmonids [8,12] and the two regions are denoted class IA and class IB [11,13]. The classical *UBA *locus in addition to a *ULA *locus lacking a transmembrane region reside in the IA region, while a class Ib pseudolocus *UCA *was found in the Atlantic salmon IB region [13]. This is in contrast to what was found in rainbow trout, where the IA region only contained a *UBA *locus and the IB region contained three expressed nonclassical genes (*UCA*, *UDA *and *UEA*) in addition to one *UFA *pseudolocus [11]. Studies by Kiryu *et al *and Miller *et al *[14,15] revealed the existence of additional MHC class I loci in salmonids (*UGA*, UHA and ZE), although the genomic location of these loci remained unclear. Additionally, a class I molecule first reported by Shum *et al*. [16], was defined as if belonging to the U-lineage (*UAA*), despite its phylogenetic divergence from traditionally U-lineage molecules. An equally divergent class I molecule defined as the L-lineage was reported by Dijkstra *et al *[3] in rainbow trout, Atlantic salmon, zebrafish and fathead minnow.

Here we describe the genomic organization of U-, Z-, and S-lineage MHC class I genes in Atlantic salmon, where SAA (S-lineage) replaces the UAA nomenclature because of its distant phylogenetic relationship to any other class I molecules. Additionally expression profiling, polymorphism, peptide binding properties and phylogenetic relationship were studied to further characterize the MHC class I molecules.

## Results and discussion

### Characterization and sequencing of BAC clones

An Atlantic salmon BAC library was screened with probes for UHA, UAA and ZE, and positive BACs were ordered into contigs using restriction fragment analysis together with GRASP HindIII fingerprint information. After PCR verification that the BACs contained sequence of interest, 184H23 and 114L13 were chosen as sequencing candidates for UHA and UAA respectively. We rename UAA to SAA (see arguments below) and will use this definition throughout the paper.

**Figure 1:**
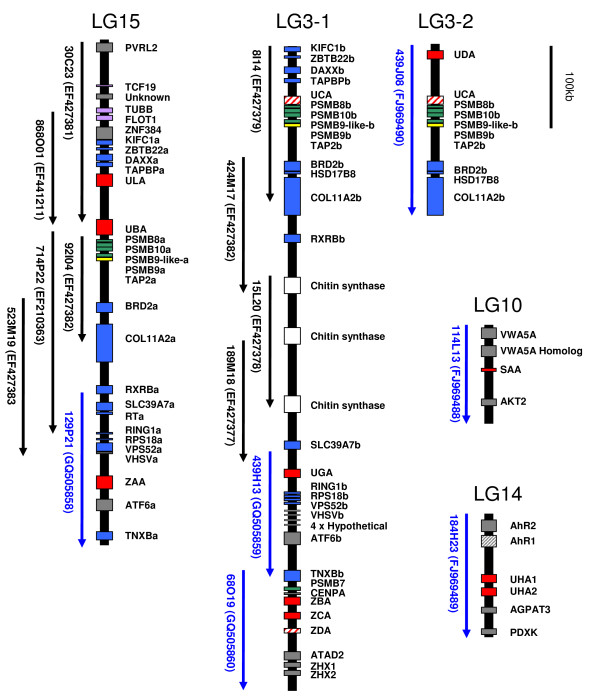
**Gene organization of Atlantic salmon MHC class I regions**. LG15 is the Atlantic salmon MHC IA region with the previous sequenced BACs 30C23, 868O01, 92I04, 714P22 and 523M19 extended with 129P21. LG3-1 is the Atlantic salmon MHC IB region with the previous sequenced BACs 8I14, 424M17, 15L20 and 189M18 extended with 439H13 and 68O19, and LG3-2 is the allelic BAC 439J08. LG10 and LG14 containing the BACs 114L13 and LG14 respectively. Genbank accession numbers are indicated after the BAC clone name. Locus designation is based on sequence identity to matching ESTs and human nomenclature is used. The regions are drawn to scale. Color code: red is MHC class I genes, yellow is TAP genes, green is proteasome genes, blue is human extended MHC class II region genes, purple is human class I region genes, grey is non-human class I region genes, pseudogenes are striped.

The ZE positive BACs clustered into two contigs and were shown to represent extensions of sequenced BACs in the duplicated MHC class I regions based on fingerprint data [13]. 129P21 was chosen from the IA region, while 68O19 for the IB region. 439H13 was later chosen and sequenced to obtain a continuous sequence of the IB region and the overlapping sequence of 439H13 and 68O19 showed 100% sequence identity over 11055 bp. An overview of the genomic position of all sequenced BACs covering the IA and IB regions is shown in Figure 1.

In our previous study we identified two BACs (439J08, 357C09) positive for an allelic version of the IB region since PCR analysis revealed that these two BAC clones possessed different UCA-like sequences [13]. Thus they were chosen as sequencing candidates. Additionally we chose to sequence a BAC that was PCR positive for *UGA *(222F07).

The BACs 439J08, 222F07, 184H23, 114L13 and 357C09 were initially sequenced using 454 technologies. Later Sanger shotgun libraries were made and sequenced to close gaps for 439J08, 114L13 and 184H23. The remaining BAC clones 129P21, 439H13 and 68O19 were subjected to Sanger sequencing only.

Our additional sequence data i.e. 129P21 extends the Atlantic salmon IA region to cover 650 kb, while the IB region being extended with 222F07, 439H13 and 68O19 now covers 870 kb. 439J08 was indeed an allelic variant of the previously IB representative 8I14, with an insertion of 50 kb that increased the total described IB region to 920 kb.

To identify the genomic location of the SAA and UHA positive BACs they were mapped in a mapping reference panel to linkage groups 10 and linkage group 14 respectively [17] (Additional File 1). The IA region containing the classical locus UBA resides on linkage group 15 while the nonclassical IB region is located on linkage group 3 [13]. An association between linkage groups and chromosomes can be found in Phillips *et al *[18].

The BAC clones were submitted to Genbank and have the following accession numbers 114L13 [134379 bp, Genbank: FJ969488], 184H23 [186322 bp, Genbank: FJ969489], 439J08 [218010 bp, Genbank: FJ969490], 68O19 [240893 bp, Genbank: GQ505860], 439H13 [152251 bp, Genbank: GQ505859] and 129P21 [202799 bp, Genbank: GQ505858]. 222F07 and 357C09 are only partially sequenced using 454 and thus not sent to Genbank.

### Genomic surroundings of Atlantic salmon U-, S-, and Z- lineage genes

We have used nomenclature with IA covering the *UBA *locus region and IB for the duplicated UCA/UDA region. New genes identified here will be named accordingly; the IA genes are given an extension of a (e.g. *RING1a*) and the IB genes have an extension of b (e.g. *RING1b*). Previously identified MHC class I molecules from the Z-lineage have been termed ZE with allelic extensions. We here propose that the four Z-lineage loci should be named with three letters as used for other MHC class I genes i.e *ZAA*; first letter - describing lineage, second letter - increasing letters A, B, C and D to reflect locus and the last letter - A for alpha chain.

A MHC class I gene residing in 114L13 was originally defined as *UAA *in rainbow trout by Shum [16]. Here we rename this gene to *SAA *to visualize the fact that this molecule shows very low identity to other U-lineage molecules (this nomenclature will also be used for the rainbow trout molecule, i.e. *Onmy-SAA*). We propose that the lineage should be designated as S as this class I lineage was first described in salmonids. The gene organization of U-, S- and Z-lineage containing regions are shown in Figure 1. 439J08 represents an allelic version of the IB region residing on LG3 thus denoted LG3-2.

Genes for *ZAA*, *ATF6a *and *TNXBa *were found in the extended IA region. The IB region was extended with genes for *UGA*, *RING1b*, *RPS18b*, *VPS52b*, *VHSVb*, *ATF6b*, *TNXBb*, *PSMB7*, *CENPA*, *ZBA*, *ZCA*, *ZDA*, *ATAD2*, *ZHX1*, *ZHX2*. We also found four almost identical genes in the extended IB region which had several EST matches [ex. Genbank: EG844714] and we define them as Hypothetical (Figure 1) as their functions are currently unknown. The three first genes were 100% identical on a protein level, while the last gene contained one amino acid substitution. Genes identified in 114L13 on LG10 were *SAA*, *VWA5A*, *VWA5A*-homolog, *AKT2*, and in 184H23 two UHA genes (*UHA1 *and *UHA2*) were identified in addition to *AhR1*, *AhR2*, *AGPAT3 *and *PDXK*.

The gene TNXB was found in both the IA and IB regions, being an Atlantic salmon ortholog of a gene residing in the extended human MHC class III region. TNXB is a member of the tenascin family of extracellular matrix glycoproteins, which has anti-adhesive effects and functions in matrix maturation during wound healing [19]. ATF6 was also identified in both regions. The human ATF6-beta (CREBL1) is located close to TNXB on chromosome 6, while ATF6-alpha is located on chromosome 1. ATF6-beta functions as a transcription factor in the unfolded protein response pathway during endoplasmatic reticulum stress [19].

We also found genes in the IB region that we have previously found in the IA region; RING1, RPS18, VPS52 and VHSV. Some of these genes were quite divergent from their IA counterparts with 91%, 100%, 75% and 70% amino acid identity respectively. *VPS52 *in the IB region is probably a pseudogene, since the gene only consisted of 293 amino acids (aa) compared to 773 aa in IA. However, *VPS52 *showed 100% identity toward a predicted CDS in Genbank [ACM08348], thus indicating that this IB genes is still expressed. *VPS52 *in humans is located on chromosome 6 in a head-to-head orientation with the gene encoding ribosomal protein S18.

The *ATAD2*, *ZHX1 *and *ZHX2 *genes identified in the extended IB region have an identical gene orientation as that found on human chromosome 8. ATAD2 belongs to a large family of ATPases and these proteins often perform chaperone-like functions that assist in the assembly, operation and disassembly of protein complexes [19]. Members of the zinc fingers and homeoboxes gene family (i.e. *ZHX1 *and *ZHX2*) are nuclear homodimeric transcriptional repressors that interact with the A subunit of nuclear factor-Y (NF-YA) and contain two C2H2-type zinc fingers and five homeobox DNA-binding domains [19].

We found a cDNA match for *PSMB7 *(proteasome subunit Z; [Genbank: BT046757]) which is a subunit of the 20S proteasome subunit complex in the IB region. Perhaps a duplicated *PSMB7 *gene exists in Atlantic salmon as another sequence [Genbank: ACI68005] showed 68% identity on the protein level. *PSMB7 *is located on chromosome 9 in humans and on chromosome 21 in zebrafish (i.e. the gene is not MHC linked) as the human and zebrafish MHC region resides on chr.6 and chr.19 respectively.

Genes identified in the UHA and SAA positive BACs were two AhR genes, *AGPAT3 *and *PDXK *in 184H23 and *VWA5A *and *AKT2 *in 114L13. The AhR2 gene shows high similarity towards a Genbank mRNA sequence ([Genbank: AY219864]; more than 99% identity), but lacks exon1 and 2. Hansson *et al *described three Atlantic salmon AhR2 genes (alpha, beta and gamma) and two AhR1 genes (alpha and beta), all containing a minimum of 11 exons [20]. Either the two lacking AhR2 exons are located upstream of 184H23 or this gene is a pseudogene with a functional copy somewhere else. We found no matching EST for the predicted AhR1 gene which we then define as a pseudogene.

*AGPAT3 *and *PDXK *have also been identified in zebrafish located on chromosome 1. *AGPAT3 *shows 83% identity to one Genbank EST [Genbank: ACI33566] whilst another EST clone [Genbank: DW540133] shows 93% identity suggestive of a duplicated locus elsewhere. This gene is highly conserved with the salmon sequence displaying more than 74% identity towards orthologs in mammals such as human, horse and pig. AGPAT3 is involved in the second step in the de novo phospholipid biosynthetic pathway where AGPAT3 is an acyltransferase that converts lysophosphatidic acid into phosphatidic acid [19]. PDXK phosphorylates vitamin B6, a step required for the conversion of vitamin B6 to pyridoxal-5-phosphate, an important cofactor in intermediary metabolism [19]. We found two copies of VWA5A, which Martin *et al *show might function as a candidate tumor supressor gene in man and the gene is located on human chromosome 11q23-q24 [21].

Table 1 shows alternative nomenclature and matching ESTs for described genes. Other open reading frames were also identified, but they were associated with transposon related repetitive elements.

**Table 1:** EST match to genes in the Atlantic salmon MHC IA and IB regions

Genes in IA/IB region	Abbreviation	Alias	EST/cDNA
MHC class I	UDA		GE768625, GE768626^(a)^

MHC class I	UGA		CB498868, EG903380

Ring finger protein 1	RING1	RNF1	GE771348, GE771349, EG819994^(b)^

Ribosomal protein S18	RPS18	KE3	EG880672

Vacuolar protein sorting 52	VSP52	SAC2	BT056476

VHSV induced gene	VHSV		BT072557

MHC Class I	ZAA		EG816925, DQ099914

Hypothetical Protein	Hypo		EG844714

Activating transcription factor 6	ATF6	CREBL1	GE795962^(b)^

Tenascin XB	TNXB	XB	n.i

Proteasome subunit, beta type, 7	PSMB7	Z	BT046757

Centromere Protein A	CENPA	CENP-A	BT048223

MHC Class I	ZBA		DY730127, EG827413

MHC Class I	ZCA		DW576043, DW559009, DW559010, DY740683

MHC Class I	ZDA		n.i

ATPase family, AAA domain containing 2	ATAD2		DY713892, DW547688, DY719530^(b)^

Zinc fingers and homeoboxes 1	ZHX1		EG895467, DW549831, DW560133, DW560132^(b)^

Zinc fingers and homeoboxes 2	ZHX2		EG815474, Eg815473, DW567814, DW566896^(b)^

			

**Genes in 184H23**			

aryl hydrocarbon receptor 2 alpha	AhR2		AY219864

aryl hydrocarbon receptor 1	AhR1		n.i

MHC Class I	UHA1		EG787974, DW548896^(a)^

MHC Class I	UHA2		GE766956, DY713347

1-acylglycerol-3-phosphate O-acyltransferase 3	AGPAT3	LPAAT-gamma	GE796418, DW540133

Pyroxidal kinase	PDXK	PNK	BT045602

			

**Genes in 114L13**			

von Willebrand factor A domain containing 5A	VWA5A	BCSC-1	DY698831, DY698832, DW563762, DW538399, DW563761

MHC Class I	SAA		DY713846, DW536069

v-akt murine thymoma viral oncogene homolog 2	AKT2		DY734945, DY698459, GO058948

Current abbreviation vs. older nomenclature is listed for each gene. All EST/cDNA sequences are from Genbank (n.i. denotes not identified). Additional comments: ^(a) ^Full-length sequencing of cDNA clones in-house (data not shown), ^(b) ^There is not sufficient EST/cDNA information to cover the entire open reading frame).

### Atlantic salmon MHC Class I genes

**Figure 2:**
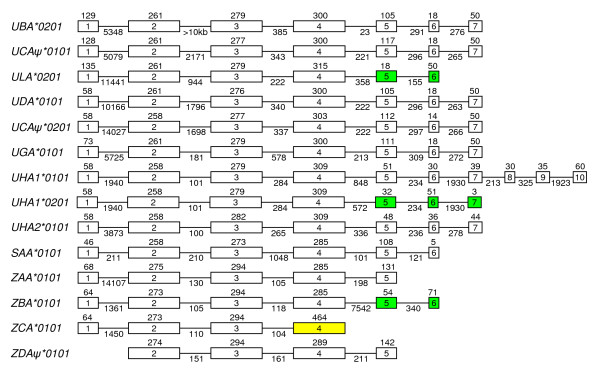
**Exon and intron boundaries for Atlantic salmon MHC class I genes from the U-, Z- and S- lineages**. The exons are boxed with the sizes in bp above sequence and intron sizes below. Green exons indicate genes that do not contain typical transmembrane and cytoplasmic domains, yellow exon in ZCA indicate merged exons of α3 domain and TM and CYT.

In the IA region only one new MHC class I locus was identified (*ZAA*). In the IB region, a functional *UDA *locus was identified 50 kb upstream of the *UCA *pseudo locus in addition to the *UGA *locus and three Z-lineage loci (*ZBA*, *ZCA *and *ZDA*). Figure 2 shows exon and intron boundaries for the described Atlantic salmon MHC class I genes from the U-, Z- and S-lineages.

### U-lineage

#### Sasa-UDA and Sasa-UCAψ

The 439J08 BAC is an allelic version of the IB region previously sequenced and represented by the BAC 8I14 [13]. Previously we identified only one MHC class I gene, the *UCA *pseudo locus in the IB region. A similar pseudo locus was also identified in the allelic 439J08 BAC showing 93% sequence identity towards the *UCAψ *in 8I14. We thus named the previously reported UCA allele UCAψ *0101, whilst the allele in 439J08 is named UCAψ *0201. The UCAψ *0201 allele contains an identical internal stop codon as in UCAψ *0101 allele making this a pseudogene as well. The UCAψ *0201 allele in 439J08 shows 93% sequence identity towards the UCAψ *0101 allele in 8I14.

439J08 contains a duplicated MHC class I locus residing approximately 50 kb upstream of UCAψ *0201, which we named *Sasa-UDA*. Dijkstra *et al*. [22] found a similar haplotype variation in rainbow trout. We found an EST match for this *UDA *locus [Genbank: GE768625], which is fully intact and expressed. The 439J08 UDA allele shows 95% sequence identity towards the UCAψ *0101 allele in 8I14. *Sasa-UDA *showed 90% identity towards rainbow trout UDA*0301 [Genbank: AY523671] and 89% identity to Onmy-UCA*0301 [Genbank: AY523661]. Only a limited polymorphism was observed in the few EST or full-length cDNAs available for UCA and UDA sequences. Further studies are needed to assess the polymorphic content for Atlantic salmon *UCA *and *UDA *loci in comparison to what Dijkstra *et al*. described in rainbow trout [22].

Comparing the region between *UCAψ *and COLLA2, there is approx 98% identity between the 8I14 (LG3-1) and 439J08 (LG3-2) sequences, while the 439J08 sequence upstream of the UCA locus has no identity to 8I14 apart from the *UDA *locus. Sequence comparison shows that there is 100% identity for the other encoded proteins in 439J08 and 8I14. The partial sequence of 357C09, being an extended version of 439J08, did contain genes for TAPBP, DAXX, ZBTB22, KIFC1, FLOT1 and TUBB upstream of the UDA locus similar to what is found in the IA region. As the 357C09 sequence consists of 20 unlinked contigs the exact organization of these genes in the IB region remains unknown.

#### Sasa-UGA

The *Sasa-UGA *locus is inserted in the IB region between the genes for SLC39A and RING1. Multiple salmon ESTs matched this locus [Genbank: CB498868 and CA043257] and assembled together (GRASP cluster 280267) they provide a full-length *Sasa-UGA *cDNA sequence with 81% sequence identity towards rainbow trout UGA [Genbank: AAP04358].

#### Sasa-UHA

The two Sasa UHA genes identified in 184H23 were named *UHA1 *and *UHA2 *and both loci have matching ESTs. The duplicated *UHA1 *and *UHA2 *loci both showed more than 93% identity towards ESTs previously named UHA and UHB by Miller *et al *[15]. A distinct difference between *UHA1 *and *UHA2 *is a three nucleotide insertion (an extra amino acid) in the α2-domain in *UHA2*. *UHA1 *and *UHA2 *are oriented in the same direction and show high similarity towards each other, but approximately 40-50% identity towards other U-linage molecules. Two different ESTs matching the *UHA1 *locus were identified, and they are most likely splice variants. UHA1*0101 [Genbank: EG787974] contains 10 exons, while UHA1*0201 [Genbank: DW548896] contains 7 exons, with the last exon containing only a stop-codon. UHA1*0201 contains an inserted exon consisting of 32 bp after the α3 domain (exon4), which contributes to an earlier stop in the CDS compared to (UHA1*0101). The nomenclature used for these splice variants is only temporary, awaiting a nomenclature debate.

### S-lineage

The *SAA *locus identified in 114L13 showed 96% aa identity to Onmy-SAA*0101 [Genbank: AAB57877], and we found a matching Atlantic salmon EST [Genbank: DY713846]. As mentioned above, the salmonid SAA sequence shows very little similarity towards other U-lineage genes, with for instance approx 30% identity to *Sasa-UBA *alleles. Sasa-SAA*0101 is a compact class I gene with small intron sizes compared to the other MHC class I genes. Sasa-SAA*0101 contains the same characteristics as Onmy-SAA*0101 [16] with six exons and five introns, and an incapacitated transposable element in intron 3.

### Z-lineage

We found 4 Z-lineage genes, one in the extended IA region and three in the extended IB region and this is in accordance with Miller *et al *[15] who identified four different ZE exon 2 sequences. The previously reported Atlantic salmon Sasa-ZE*0101 sequence [Genbank: DQ099914] shows 91% sequence identity towards the IA linked *ZAA *locus, and most likely emerged from this locus. The *ZAA *sequence in the 129P21 BAC is named Sasa-ZAA*0201, while the previously reported sequence Sasa-ZE*0101 should be renamed Sasa-ZAA*0101.

We have also identified matching EST clones for *ZBA *[Genbank: DY730127 and EG827413] and *ZCA *[Genbank: DY740683 and DW559009] making these bona-fide genes, while *ZDA *lacks the leader peptide suggestive of a pseudogene. ZBA*0101 and ZCA*0101 show 96% sequence identity over 918 bp, while the remaining 3' sequence shows little sequence identity.

Comparing the separate exons of all the Z-lineage genes (*ZAA, ZBA, ZCA*, *ZDAψ*), the highest sequence identity overall was found in exon2 and exon3, ranging from 89-96% identity for exon2, 84-92% identity for exon3, while exon4 showed 69-86% identity (data not shown). Comparing the different exons of ZBA*0101 and ZCA*0101 we found 95%, 92% and 86% identity in exon2, exon3 and exon4 respectively. They are all orientated in a head to tail fashion.

The exon-intron organization of the four Z-lineage MHC class I genes are quite different with 5, 6, 4 and 4 exons for *ZAA*, *ZBA*, *ZCA *and *ZDAψ *respectively. The connecting peptide, transmembrane and cytoplasmic tail domains are all encoded by exon 5 in *ZAA*. In *ZBA*, this region in encoded by exons 5 and 6 where the transmembrane region has been lost. In *ZCA*, the 4^th ^exon is larger than for any other class I gene and encodes the α3 domain in addition to the connecting peptide, transmembrane and cytoplasmic region. However this exon-intron organization is truly unorthodox. A sequence comparison of the *ZBA *sequence with the genomic region downstream of the *ZCA *α3 domain showed that a similar exon-intron organisation as in *ZBA *could be identified for *ZCA *as well. Thus the ESTs identified for the *ZCA *gene could be splicoforms.

### Phylogenetic analysis of fish MHC class I molecules

**Figure 3:**
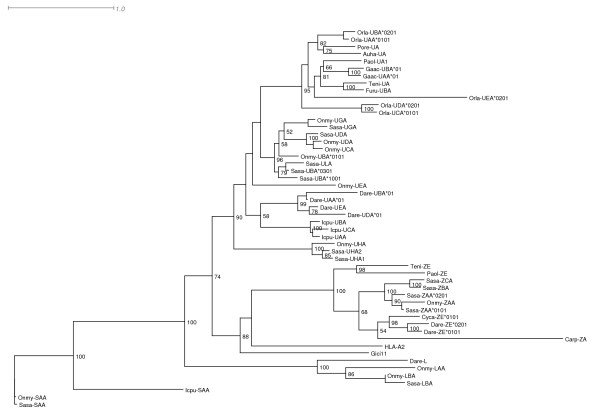
**Unrooted Phylogenetic tree of teleost MHC class I sequences**. Phylogenetic tree analysis performed using PhyML 3.0 based on JTT model of evolution for full-length amino acid sequences. Consensus trees were based on 100 bootstrap replications and reported with the bootstrap support values (in percent) indicated at the respective nodes. Sequence references are as follows:Auha-UA [Genbank: AAD37813], Dare-UBA*01 [Genbank: CAA86732], Dare-UAA*01 [Genbank: CAA86731], Dare-UDA*01 [Genbank: AAF20178], Dare-UEA [Genbank: AAH53140], Dare-ZE*0201 [Genbank: CAD12790], Dare-L [Genbank: CAD56801], Furu-UBA [Genbank: AAC41236], Gaac-UAA*01 [Genbank: ABN14358], Gaac-UBA*01, Genbank: ABN14357], Icpu-SAA [Genbank: CK423282], Icpu-UAA [Genbank: AAD08650], Icpu-UBA [Genbank: AAD08648], Icpu-UCA [Genbank: AAD08647], Onmy-SAA [Genbank: AF091779], Onmy-UBA [Genbank: AF287483], Onmy-UCA [Genbank: BAD89552], Onmy-UDA [Genbank: AY523666], Onmy-UEA [Genbank: BAD89553], Onmy-UGA [Genbank: AAP04358 ], Onmy-LAA [Genbank: ABI21842 ], Onmy-LBA [Genbank: ABI21844], Orla-UAA*0101 [Genbank: BAD93265], Orla-UBA*0201 [Genbank: BAB83850], Orla-UCA*0101 [Genbank: BAB63957], Orla-UDA*0201 [Genbank: BAB83843], Orla-UEA*0201 [Genbank: BAB83837], Paol-UA1 [Genbank: BAD13367], Paol-ZE [Genbank: BAD13366], Pore-UA [Genbank: CAA90791], Sasa-LBA [Genbank: DY733800 and GO062643], Sasa-UBA*0301 [Genbank: AAN75116], Sasa-UBA*1001 [Genbank: AAN75118 ], [Genbank: ABQ59666], Sasa-ZAA*0101 [Genbank: DQ099914], Teni-UA [Genbank: CR724171], Teni-ZE [Genbank: CAF90807], HLA-A2 [Genbank: AAA76608]. Sasa-UDA, Sasa-UGA, Sasa-UHA1, Sasa-UHA2, Sasa-ULA, Sasa-SAA, Sasa-ZAA*0201, Sasa-ZBA, Sasa-ZCA are described in this paper.

The MHC class I amino acid sequences from the U-, S-, Z- and L-lineages in addition to MHC class I sequences from various fishes, man and shark were used to draw a phylogenetic tree (Figure 3 and Additional File 2). With bootstrap values ranging from 90-100% the U, Z, L and S sequences form distinct clusters. Most published fish class I sequences belong to the U-lineage, while the Z-lineage so far contains sequences from fewer teleost species including the pufferfish family, zebrafishes, carp and Japanese flounder. L-lineage class I molecules are so far only identified in zebrafish, fathead minnow and salmonids, while class I sequences from the S-lineage are only found in catfish and salmonids.

Among the Atlantic salmon U-lineage class I sequences there is a close relationship between the *Sasa-UBA *and *Sasa-ULA *suggesting that *ULA *is a recent duplication of *UBA*. An *ULA *homolog is so far not identified in rainbow trout. *Sasa-UDA *and *Sasa-UGA *cluster on separate nodes together with their rainbow trout homologs. Overall, the U-lineage sequences from all teleosts cluster together with their taxonomic superorder families, that is; acanthopterygii, protacanthopterygii and ostariophysi. One exception is the *Sasa-UHA1 *and *Sasa-UHA2 *that branch off early on a separate node from all other U-lineage sequences, showing a more distant phylogenetic relationship. Whether the UHA sequences diverged this early as the phylogenetic tree indicates stands as an open debate as the UHA genes are located on a separate linkage group (LG14), suggesting a separate evolutionary origin from the other U-lineage located on linkage group 3 and 15. Interestingly, the α3 domain (exon 4) of the UHA molecules shows very low amino acid conservation in comparison to other U-lineage genes. Likewise, the human nonclassical gene MR1 [23], show a similar identity in the α1 and α2 domains towards the classical counterparts (HLA-A, HLA-B and HLA-C) while the α3 domain is more divergent. The functional relevance of this lack of conservation remains unknown.

The Z-lineage molecules also cluster together with their taxonomic superorder families, although including fewer sequences than the U-lineage sequences. So far the Atlantic salmon is the only teleost species reported, with 3 different expressed Z-lineage genes.

The Atlantic salmon S-lineage molecule shows a close relationship to the *Onmy-SAA *molecule. Based on their sequence identity, both molecules seem to have evolved very little following the divergence of these two species [24].

### Expression of Atlantic salmon MHC class I genes

**Figure 4:**
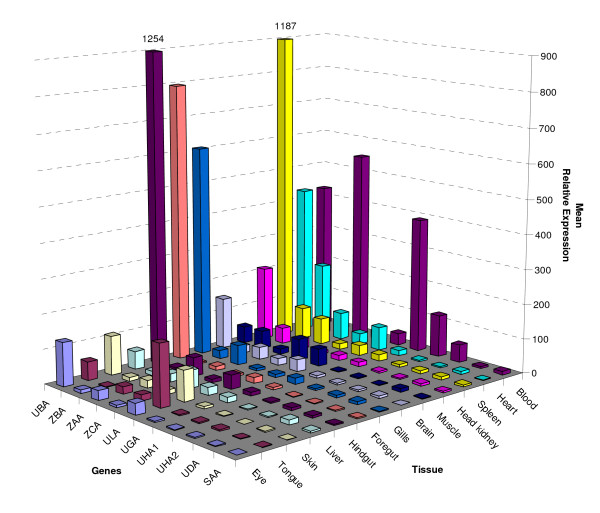
**Expression analysis of MHC class I genes in Atlantic salmon**. Relative expression of *Sasa-UBA*, *Sasa-UDA*, *Sasa-UGA*, *Sasa-UHA1*, *Sasa-UHA2*, *Sasa-ULA*, *Sasa-ZAA*, *Sasa-ZBA*, *Sasa-ZCA and Sasa-SAA *in various tissues of Atlantic salmon using EF1A as reference gene. The Relative expression values for UBA in hindgut and spleen has been truncated and their values are indicated above their respective bars.

One of the definitions of a MHC class Ia gene is that it is expressed in most tissues. To investigate the expression patterns of Atlantic salmon MHC class I molecules, blood, foregut, hindgut, head kidney, gills, spleen, liver, eye, tongue, skin, muscle, heart and brain samples were taken from three Atlantic salmon individuals and analyzed for gene expression by quantitative real-time PCR (Figure 4). To evaluate the expression patterns from the heart, blood was included as sample to differentiate between the expression patterns in heart structural tissue.

Highest expression for *UBA *was observed in foregut, hindgut, spleen and gills, while moderate expression was observed in blood and heart. Lowest expression was observed in tongue, liver and brain. Without using an absolute quantification method i.e. including a standard curve, it is not possible to compare different genes for same tissues, however the transcriptional level of other MHC class I genes were low in comparison to *UBA *with the exception of *UGA *in blood. In general, the high expression of *UBA *in comparison to the other MHC class I genes, is also supported by hits found in databases. Various BlastN, BlastX, and TBlastN searches in Genbank [25] and cGRASP [26,27] using UBA sequences hit numerous sequences, while blasting with other U-, S-, or Z-lineage sequences results in generally few hits.

We found that *ULA *had highest expression in tongue, and lower in skin and heart. In contrast to our results, Miller *et al *[15] found that *ULA *was only weakly expressed in posterior kidney in un-infected fish, while expression was up-regulated in IHNV-infected liver, brain and eye tissues. They did not detect expression in the heart of un-infected nor infected tissues. *UDA *showed little variation in expression patterns between the different tissues tested. The remaining nonclassical U-lineage class I genes, *UHA1*, *UHA2 *and *UGA *all showed the highest expression in blood.

Expression analysis of the Z-lineage genes showed that *ZAA *had highest expression in blood, while lower expression in the heart, spleen, gills and hindgut. *ZBA *was 2-fold more expressed in the heart than in the spleen, but lower in the other tissues. The Z-lineage genes seem ubiquitously expressed in most tissues tested similar to that observed by Miller *et al *[15]. Little difference in expression patterns for the various tissues was observed for the *SAA *gene.

Both *ULA *and *ZBA *lack transmembrane, connecting peptide and cytoplasmic domains and probably are secreted MHC class I molecules. Based on their expression patterns they could have specific functions in the heart. The Z-lineage expression such as *ZAA *in blood and heart, and *ZBA *in the heart, suggest interesting functions to be unravelled.

During the initial studies we checked the PCR products amplified during realtime PCR on agarose gels, and observed that products for *UHA1 *contained two bands that were equally expressed, with exception in muscle where the smallest band was missing. Sequencing of these PCR products showed that the largest fragment represented the UHA1*0201 allele with the additional exon, while the smallest PCR product represented the UHA1*0101 allele.

We have only investigated un-stimulated tissues, and it is most likely that the expression patterns would be different in stimulated tissues. Based on expression patterns and polymorphic content only *UBA *can be classified as a true classical MHC class I gene, while the others should be classified as nonclassical MHC molecules.

### Structure and function of Atlantic salmon MHC class I molecules

**Figure 5:**
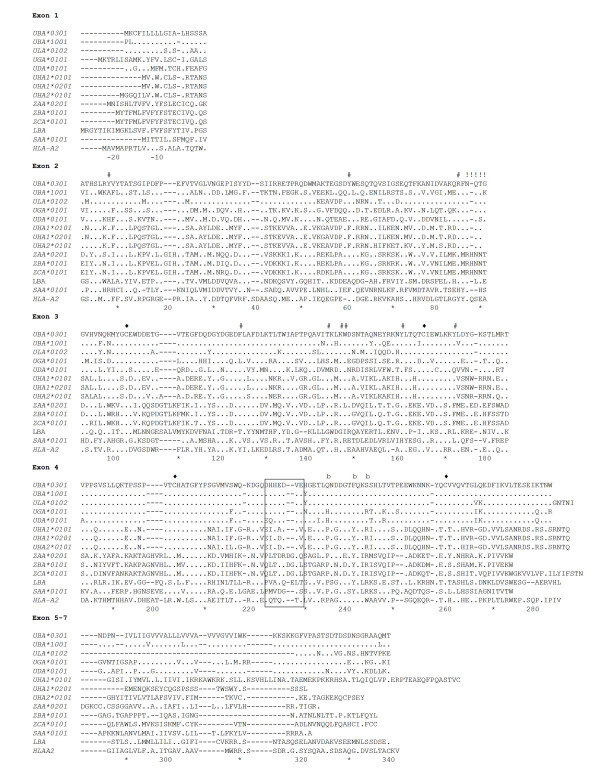
**Alignment of Atlantic salmon MHC class I sequences**. Comparison of Atlantic Salmon UBA*0301 [Genbank: AAN75116], UBA*1001 [Genbank: AAN75118], *ULA *[Genbank: ABQ59666], *UGA*, *UDA*, *UHA1*, *UHA2*, *ZAA*, *ZBA*, *ZCA*, *SAA *(described in this paper), LBA [Genbank: DY733800 and GO062643] and HLA-A2 [Genbank: AAA76608]. Dots indicate identities, dashes indicate gaps or missing sequence information. Cysteine residues involved in putative Ig fold are marked ♦ above sequences. Anchor residues known to bind the ends of peptide are marked # based on mammalian positions. Unique N-linked glycosylation sites are marked ! and CD8 binding site are boxed based on the acidic stretch from mammalian. b defines Z, S, L and UHA residues potentially involved in interactions with a different beta2-microglobulin. Individual domains/exon for LBA are based on the Sasa- UBA*0301 sequence.

Structure and composition of MHC class I genes are quite variable both within and between vertebrate groups, with the α1α2 domains containing most of the polymorphism. In mammals, the presence of nine evolutionary conserved sites within the α1α2 domains have been defined as residues that anchor the peptide [28,29]. These residues are highly conserved in classical as well as in most nonclassical molecules like HLA-E, HLA-F and HLA-G. The set of residues in mammals are YYYYYYTKW, while in non-mammalian vertebrates it has changed slightly to YYYYRFTKW [16,23]. Deduced Atlantic salmon MHC class I amino acid sequences identified in our BACs in addition to a sequence belonging to the L-lineage (LBA) [Genbank: DY733800 and GO062643] were aligned with human MHC class I sequences (Figure 5). Presence of conserved peptide anchoring sites for all Atlantic salmon class I sequences including human, chicken and shark are summarized in Table 2.

**Table 2:** Comparison of nine conserved residues involved in peptide binding

	**Peptide N terminus**				**Peptide C terminus**					
			
	**7**	**59**	**159**	**171**	**84**	**123**	**143**	**146**	**147**	**Diff.**
		
UBA*0301	Y	Y	Y	Y	R	F	T	K	W	
UBA*1001	A	-	-	-	-	-	-	-	-	1
ULA	-	-	-	-	-	-	-	-	-	0
UGA	-	-	-	-	-	L	S	-	-	2
UDA	H	-	-	Q	-	-	M	-	-	3
UHA1	-	F	-	-	-	-	-	-	-	1
UHA2	-	F	-	-	-	-	-	-	-	1
ZAA	-	-	-	F	-	-	-	-	L	2
ZBA	-	-	-	F	-	-	-	-	-	1
ZCA	-	-	-	F	-	-	-	-	-	1
SAA	R	F	H	L	H	-	Y	-	R	7
LBA	A	E	V	I	H	A	Q	D	G	9
B-F19	-	-	-	-	-	-	-	-	-	0
Trsc-UAA	-	-	-	-	-	L	-	-	-	1
HLA-A2	-	-	-	-	Y	Y	-	-	-	2

Comparison of Atlantic Salmon UBA*0301 [Genbank: AAN75116], UBA*1001 [Genbank: AAN75118], *ULA *[Genbank: ABQ59666], *UGA*, *UDA*, *UHA1*, *UHA2*, *ZAA*, *ZBA*, *ZCA*, *SAA *(described in this paper), LBA [Genbank: DY733800 and GO062643], B-F19 (Chicken) [Genbank: M84766], *Trsc*-UAA*101 (Shark) [Genbank: AF034316] and HLA-A2 [Genbank: AAA76608].

For the MHC class I *UBA *locus we included two highly divergent alleles, where UBA*0301 contains the non-mammalian motif while UBA*1001 differ in one residue, A7. One or rarely two substitutions of these conserved residues have also been observed in both human and mouse MHC class Ia sequences [28,29]. The *ULA *locus described as a secretory class I molecule due to a missing transmembrane domain [13], does display the conserved motif for non mammalian vertebrates, and may thus still bind peptides. The two UHA loci have one residue different from the classical *UBA *locus at F59. A tyrosine/phenylalanine substitution is common amongst non mammalian vertebrates, and may not interfere with binding capacity. The *UDA *and *UGA *have two and three residues different from *UBA*. These are not conservative substitutions (e.g. Y/F), and most likely influence peptide-binding. Overall it seems like all the U-lineage sequences in Atlantic salmon are able to present peptides based on these nine anchor residues.

Both *SAA *and *LBA *sequences have quite different residues in some or all of these positions when compared against the *UBA *classical class I sequences. Seven of the nine residues are found to differ from the nonmammalian motif in *SAA *sequences, while all of the nine residues are found to differ in *LBA *sequences. In mammals, the class Ib molecule CD1, known to bind lipid and glycolipid ligands do not contain this peptide binding motif [2]. Thus it is likely that *SAA *and *LBA *binds non-peptide ligands or none at all as the mammalian class Ib molecules MIC-A/B and HFE. In rainbow trout, Shum *et al *concluded that *Onmy-SAA *(similar to *Sasa-SAA*) has properties indicative of a particularly divergent class Ib gene [16].

The Z-lineage sequences also follow the non mammalian motif with the exception of one common different residue, F171. Three amino acids downstream of F171, all Z-lineage sequences have a tyrosine (Y) residue which might function as the conserved peptide anchoring residue. This has also been observed in cyprinid class I ZE sequences which suggests that use of the tyrosine Y174 might affect and extend the peptide binding groove, leading to binding of somewhat larger peptides [5]. Another possibility is that a substitution of a tyrosine (Y) residue by a phenylalanine (F) in position Y171 may not have major implications for the ability to bind peptide termini since most non mammalian MHC class Ia sequences have a similar substitution of a tyrosine residue by a phenylalanine residue at position Y123.

In humans there is a stretch from aa 231-244 in the class I α3 domain known to interact with β2 m [30]. In this region, the UHA and all Z-, S- and L-lineage sequences have a specific motif, P235, Y241 and R244, which may influence the association with β2 m. Atlantic salmon express up to twelve different β2 m molecules (unpublished data), which group into two lineages, BA1 [Genbank: AF180478] and BA6 [Genbank: AF180484] which may stabilize different MHC class I sequences; one lineage may bind to UHA, Z-, S- and L -lineage molecules and the other β2 m lineage may bind and stabilize the remaining U-lineage molecules that also show a conservation in this region. In humans, there are also at least four class Ib molecules that do not bind β2 m (MICA/B, EPCR, ULBP and ZAG) [2]. Future studies are needed to clarify β2 m binding of salmonid MHC class I molecules.

Other features that are shared between the Atlantic salmon class I molecules and nearly all MHC class Ia and Ib molecules of other vertebrates, include 4 conserved cysteine residues located in the α2α3 domains forming disulfide bonds within theses domains and the highly conserved FYP motif at positions 208-210 aa in the α3 domain [28]. Most class I sequences contain an N-linked glycosylation site near the end of the α1 domain. The amino acid residues which correspond to the CD8 binding site of the HLA-A locus are at positions 223-229 in the Atlantic salmon sequences. Both classical and some nonclassical mammalian MHC class I molecules have a well conserved negatively charged CD8 binding site in the α3 domain (ca.218-226). Here the Atlantic salmon classical *UBA *molecules have an acidic stretch consisting of 4 negatively charged aa (D or E), also found in the *ULA*, *UGA *and *UDA *molecules. The *UHA1 *and *UHA2 *molecules consist of 2 and 3 negatively charged aa respectively. The *SAA *molecule has 1, while the Z molecules has from 2 to 3 negatively charged aa in the this region. Both CD8α and CD8β chains have been described in the Atlantic salmon [31]. The CD8 molecule can be expressed both in a homodimeric and heterodimeric form, where the latter is most expressed in mammals, preferably in circulating lymphocytes. The lower acidic content in this region for the UHA, *SAA *and Z molecules may influence the lymphocyte interactions in contrast to the higher acidic content for *UBA*, *ULA*, *UGA *and *UDA *molecules.

Having classified the Atlantic salmon class I molecules into functional categories based on traditional criterions, the functional properties of these molecules still remain unclear. *Sasa-ULA *and *Sasa-ZBA *exists in a soluble form, while the *Sasa-UHA1 *locus expresses both membrane bound and a soluble variant as a result of alternative splicing, leading to loss of the Tm/Cyt domain by a frame shift. In humans, both MHC class Ia and Ib molecules are also known to exist in soluble forms. The classical HLA-A, -B and -C loci express soluble variants that play a role in cell death of activated T cells [32]. Another example is the human non-classical HLA-G molecules that exist in several different isoforms both membrane bound and secreted forms where the soluble variant is caused by alternative splicing where the Tm/Cyt domains are deleted [33]. The soluble HLA-G is found expressed specifically in placental tissue and is secreted during pregnancy [34] and also thought to be involved in inducing apoptosis in activated maternal CD8+ T cells [35]. The functional relevance of soluble class I molecules remain to be established in fish.

## Conclusion

Here we describe the genomic organization of U-, Z-, and S-lineage MHC class I molecules in Atlantic salmon. Nine of the described class I genes are located in the extension of the duplicated IA and IB regions, while three class I genes are found on two separate linkage groups. The gene organization of the two regions indicates that the IB region is evolving at a different pace than the IA region. The IB region contains 3 chitin synthase genes not present in the IA region. Four gene duplications are seen for both a hypothetical protein as well as for the Z-lineage genes, a phenomenon not found in the IA region. There is also haplotypic *UCA/UDA *variation while the *UEA *and *UFA *genes residing in the rainbow trout IB region have been lost since *Oncorhynchus *and *Salmo *split, which probably was around 15-20 mya. The functional consequence of this evolution remains to be established. Expression profiling, polymorphic content, peptide binding properties and phylogenetic relationship show that Atlantic salmon has only one expressed MHC class Ia gene (*UBA*), in addition to a multitude of nonclassical loci from the U-, S and Z-lineages. Further studies are needed to verify the functional properties of the MHC class Ib molecules in teleost.

## Methods

### BAC library screening

An Atlantic salmon CHORI-214 bacterial artificial chromosome (BAC) library was obtained from BACPAC Resources, Children's Hospital Oakland Research Institute [36]. The library consisted of approximately 299,000 recombinant clones, representing 18-fold genome coverage and an average insert size of 188 kb [37]. Putative full length cDNA sequence information for the *S. salar *SAA, UHA and ZE genes were obtained from GRASP EST clustering database [26,27] and used for primer design. Probes specific for the SAA, UHA and ZE genes were PCR amplified (primers listed in Table 3) from a Head Kidney cDNA pool, purified from agarose gel slices with the GeneClean III Kit (Qbiogene) and verified by sequencing. Probes were radioactive labelled with α^32^P-CTP (Amersham) using Rediprime Random Labelling Kit (Amersham), including spermine precipitation of labelled DNA. Filter hybridizations were conducted as described by CHORI. Probed BAC library filters were stored in Phospho-image cassettes for 2-24 hours and hybridizations visualized by a Typhoon Phospho Image Scanner (Amersham).

**Table 3:** Primers used for probes, screening and real time PCR

	Sequence (5'-3')	Usage
SAA_387F^1^	TATGAGCCATGCATATGAC	cDNA amplification of SAA

SAA_815R^1^	CTGTAGCTCTGGGTGTCCT	cDNA amplification of SAA

UHA_329F^1^	TGCAGAAAATGTACAGCTG	cDNA amplification of UHA

UHA_798R^1^	GTAGTTGTGCTGCTGTAGG	cDNA amplification of UHA

ZE_481F^1^	TTGAAATTCATAAAGGGCAC	cDNA amplification of ZE

ZE_909R^1^	GGGATCTGCACACTCATC	cDNA amplification of ZE

114I13-F^2^	TGATTCCCATCTCAGTATCC	Marker Ssa10083BSFU in BAC 114L13

114I13-R	TAGCTACCTTTCTGAGCCTG	Marker Ssa10083BSFU in BAC 114L13

184H23-F^2^	CAGGCCTTTACTCTCTGCTA	Marker Ssa10084BSFU in BAC 184H23

184H23-R	GGGAGTGTTGTCTAAACCTG	Marker Ssa10084BSFU in BAC 184H23

21M13	TGTAAAACGACGGCCAGT	

UBA-F	CTGACAACTCTGGGAGAGCT	Realtime PCR *Sasa-UBA*

UBA-R	GTGTGTTATGTTCTTGAGACGT	Realtime PCR *Sasa-UBA*

439_UDA_967F	CCATCATTGTCCCCATCATTG	Realtime PCR *Sasa-UDA*

439_UDA 1094R	AGATCTTTCCCAGAGTACACA	Realtime PCR *Sasa-UDA*

UGA-F	GTGAATGATCAGGTCATTAGCC	Realtime PCR *Sasa-UGA*

UGA-R	CTCTGCGCCTCTCAGGTCC	Realtime PCR *Sasa-UGA*

UHA1_800F	TACAGCAGCACAACTACACAT	Realtime PCR *Sasa-UHA1*

UHA1_920R	TGACCAATACCAAGACCATGTAGA	Realtime PCR *Sasa-UHA1*

UHA2end-F	TACACATGCACTGTCCAACAC	Realtime PCR *Sasa-UHA2*

UHA2end-R	GCCAATGTGACCAATACAATGG	Realtime PCR *Sasa-UHA2*

ULA_961F	TCAAGACCAACTGGGGAAACA	Realtime PCR *Sasa-ULA*

ULA_1094R	CCGTCTTTCTTCTCTTGTCTG	Realtime PCR *Sasa-ULA*

ZAA_847F	CCAGAGGCAGACAAGGAAAC	Realtime PCR *Sasa-ZAA*

ZAA 954R	CAGCCCCAATGACTACAGCA	Realtime PCR *Sasa-ZAA*

ZBA_840F	CCCAGAGGCAGACAAGGACA	Realtime PCR *Sasa-ZBA*

ZBA_949R	AATTAGAGAGGCCTGGATCCC	Realtime PCR *Sasa-ZBA*

ZCA_842F	CAGAGGCAGACAAGCAAACC	Realtime PCR *Sasa-ZCA*

ZCA_963Rseq	ATTGATAACCATGCAAATAACTGG	Realtime PCR *Sasa-ZCA*

SAA_379F	CCTTTATGAGCCATGCATATGA	Realtime PCR *Sasa-SAA*

SAA_504R	AATGAATGACCAGCCTAACAAG	Realtime PCR *Sasa-SAA*

^1 ^Primer design for probes for SAA, UHA and ZE is based on DW536070, DW548896 and DQ099914 respectively

^2 ^The forward primer had the 21M13 primer added to its 5'-end

### Characterization of BACs

SAA, UHA and ZE positive BAC clones were ordered into contigs by GRASP HindIII fingerprint information [38]. Selected BAC clones were picked from each fingerprint contig to represent core -and flanking BACs from each contig, followed by BAC DNA isolation described by CHORI. Positive BAC clone DNA from selected BACs were then PCR amplified with the SAA, UHA, ZE primers (Table 3) to verify that these BACs contained sequence of interest.

### 454 shotgun pyrosequencing

The shotgun sequencing protocol using the 454 sequencing system (454 Life Science, USA) was performed according to manufactures protocol. Briefly, to generate the GS FLX shotgun library, the isolated Atlantic salmon BAC DNA was sheared into fragments, to which process specific A and B adaptors were blunt end ligated. After adaptor ligation, the fragments were denatured and clonally amplified via emulsion PCR, thereby generating millions of copies of template per bead. The DNA beads were then distributed into picolitre-sized wells on a fibre-optic slide (PicoTiterPlate™), along with a mixture of smaller beads coated with the enzymes required for the pyrosequencing reaction. The four DNA nucleotides were then flushed sequentially over the plate. Light signals released upon base incorporation were captured by a CCD camera, and the sequence of bases incorporated per well was stored as a read. DNA extractions were performed in our lab, while library generation and sequencing were performed at Royal Institute of Technology, Dept of Biotechnology (Stockholm, Sweden) and CEES, University of Oslo (Oslo, Norway). Assembly of the 454 reads were performed with Newbler.

### BAC shotgun library and sequencing

The selected BACs were subjected to a shotgun sequencing approach and procedure is described in [13]. After sequencing run, bases were called using Phred [39,40]. High quality sequencing reads were assembled using Phrap [41], and viewed and edited using Consed [42]. Autofinish [43] was used for closing gaps by designing gap-closing primers with subsequent direct sequencing on BAC DNA or PCR amplification and PCR product sequencing. The BAC sequences were submitted to Genbank and given the following accession numbers: 114L13 [Genbank: FJ969488], 184H23 [Genbank: FJ969489], 439J08 [Genbank: FJ969490], 68O19 [Genbank: GQ505860], 439H13 [Genbank: GQ505859] and 129P21 [Genbank: GQ505858].

### Bioinformatics

DIGIT [44] and GENSCAN [45] were used to predict novel genes and to identify open reading frames. Dotter [46] was used to compare the BAC sequence to itself as well as to other BACs and to identify duplicated regions. Vista was used for sequence comparisons [47]. Blast searches identified possible functions of predicted genes [48,49]. Sim4 [50] and Spidey [51] were used to adjust exon and intron boundaries aligning EST/cDNA sequences [26,27] to the BAC sequences. GRASP repeatmasker was used to identify repeats [52,53]. Multiple sequence alignments of the assumed or verified expressed exons were done using ClustalX [54] followed by manual inspection. TMHMM Server v. 2.0 was used for prediction of transmembrane domains for the MHC class I genes [55-57].

The amino acid sequences were aligned using Clustal W and inferred by ProTest [58] to find the best-fitting model of evolution (JTT, with an estimated alpha parameter to 1.827, a gamma distribution of rates between sites of 4.0 and a proportion of Invariable Sites of 0.012). Phylogenetic analysis was performed using PhyML 3.0 [59], a fast and accurate maximum likelihood heuristic method, starting from the BIONJ tree under the parameters estimated by ProtTest. Tree stability was assessed by means of a bootstrap analysis with 100 cycles. A phylogenetic tree was also created using neighbour-joining method in MEGA version 4 [60]. Consensus trees were based on 1000 bootstrap replications and reported with the bootstrap support values (in percent) indicated above the respective nodes. Gaps were removed and phylogenetic data reported using the Poisson correction model with uniform rates across all sites.

### Mapping the BACs 114I13 and 184H23 in Atlantic salmon

The sequences of the BACs 114I13 and 184H23 were screened for suspected and known repetitive elements in salmonids using a salmonid-specific repeat masker [52]. The resulting sequences were subsequently searched for the presence of microsatellites using a Perl script created in Davidson Lab. 21 and 17 microsatellites were identified in 114I13 and 184H23, respectively. Primer3 software [61] was used to design primers that would amplify the microsatellites containing the largest number of repeats. Several of these primer pairs were tested on the parents of the Atlantic salmon SALMAP mapping families, Br5 and Br6 [62,63], to determine which are informative. PCR amplifications were carried out in 6 uL reaction volumes in thin walled tubes using a Biometra T3 or T3000 Thermocycler. The reaction contained: 20 ng DNA, 1 × PCR buffer, 0.05 mM dNTP, 0.2 μM of the forward primer, 0.5 μM of the reverse primer, 0.5 μM of -21M13 primer (5'-TGTAAAACGACGGCCAGT-3') labeled with HEX- or 6-FAM at the 5' end, and 0.25 U of Taq polymerase (Qiagen). Touchdown PCR was performed as follows: a 2 min initial denaturation step at 94°C, then a cycle consisting of a denaturation step of 94°C for 30 seconds, annealing for 30 seconds and an elongation step at 72°C for 30 seconds. The initial annealing temperature of 60°C was decreased by 0.5°C every cycle to 50°C, and then held at 50°C for an additional 14 cycles. The amplification products were analyzed using an ABI 377 DNA sequencer. The inheritance patterns of two of these microsatellite markers, Ssa10083BSFU from 114I13 and Ssa10084BSFU from 184H23, were examined in the SALMAP mapping families. Pair-wise linkage analysis was performed using the LINKMFEX software package with a LOD score 4 threshold [64]. Table 3 show the primer sequences of genetic markers used to place 114I13 and 184H23 on the Atlantic salmon linkage map [17].

### Gene expression analysis

The study was conducted in agreement with the provision enforced by the National Animal Research Authority (NARA) [65]. mRNA was extracted from Atlantic salmon tissues of 3 fishes (foregut, hindgut, head kidney, gills, spleen, liver, eye, tongue, skin, muscle, heart, brain and blood) using QuickPrep *micro *mRNA Purification Kit (GE Healthcare Life Science). 1 μl of mRNA sample was used for quantification with Nanodrop spectrometer (Nanodrop Technologies, DE). All samples were DNase treated using Turbo DNA-free™ (Ambion, Austin, TX, USA). Gene specific PCR primers (Table 3) were designed manually for the amplification of approximately 100-150 bp fragments and synthesized by ProOligo (Paris, France). The amplicons were, when possible, placed over introns and product size and specificity was confirmed by agarose gel electrophoresis (Gel logic 200 Imaging system, Kodak) and sequencing. cDNA synthesis were performed with Ready-To-Go T-Primed First Strand Kit (Amersham, USA) Quantitative real-time PCR was conducted on an ABI7700HT (Applied Biosystems, USA). Reactions were performed in 20 μl including 1 μl cDNA (~6 ng of mRNA) using PowerSYBR Green PCR Master Mix according to the manufacturer's instructions (Applied Biosystems). PCR parameters were 95°C for 10 min, followed by 40 cycles consisting of 95°C for 15 s, 60°C at 30 s and 72°C at 60 s. A dissociation analysis was performed for each sample to check for unspecific amplification. Relative expression of mRNA in relation to the housekeeping gene elongation factor 1α (EF1A) was calculated using the ΔC_T _method [66]. Data from real-time RT-PCR are presented as the Mean Relative Expression by calculation of mean 2^-ΔCT ^× 100 for the three fishes used, with three independent samples from each tissue from each fish.

## Authors' contributions

MFL and HH: Performed library screening and BAC restriction mapping, sequencing, sequence data analysis, annotations, real time expression and drafted the manuscript; HGB, MBS and LM: Performed library screening and sequencing; KPL: Design of the primers and mapping of BAC clones to linkage groups; BFK: Contributed to the planning and directions; UG: Contributed to planning, design, direction and analysis; All authors read and approved the final manuscript.

## Supplementary Material

Additional file 1 **Linkage groups of SAA and UHA**. The positions of SAA (Ssa10083BSFU) and UHA (Ssa10084BSFU) in bold and underlined on linkage group 10 and linkage group 14, respectively of the merged Atlantic salmon female map [17]. Click here for file

Additional file 2 **Phylogeny of teleost MHC class I sequences**. Phylogenetic tree analysis by NJ method for full-length amino acid sequences. Consensus trees were based on 1000 bootstrap replications and reported with the bootstrap support values (in percent) indicated above the respective nodes. Sequence references are as follows:Auha-UA [Genbank: AAD37813], Dare-UBA*01 [Genbank: CAA86732], Dare-UAA*01 [Genbank: CAA86731], Dare-UDA*01 [Genbank: AAF20178], Dare-UEA [Genbank: AAH53140], Dare-ZE*0201 [Genbank: CAD12790], Dare-L [Genbank: CAD56801], Furu-UBA [Genbank: AAC41236], Gaac-UAA*01 [Genbank: ABN14358], Gaac-UBA*01, Genbank: ABN14357], Icpu-SAA [Genbank: CK423282], Icpu-UAA [Genbank: AAD08650], Icpu-UBA [Genbank: AAD08648], Icpu-UCA [Genbank: AAD08647], Onmy-SAA [Genbank: AF091779], Onmy-UBA [Genbank: AF287483], Onmy-UCA [Genbank: BAD89552], Onmy-UDA [Genbank: AY523666], Onmy-UEA [Genbank: BAD89553], Onmy-UGA [Genbank: AAP04358 ], Onmy-LAA [Genbank: ABI21842 ], Onmy-LBA [Genbank: ABI21844], Orla-UAA*0101 [Genbank: BAD93265], Orla-UBA*0201 [Genbank: BAB83850], Orla-UCA*0101 [Genbank: BAB63957], Orla-UDA*0201 [Genbank: BAB83843], Orla-UEA*0201 [Genbank: BAB83837], Paol-UA1 [Genbank: BAD13367], Paol-ZE [Genbank: BAD13366], Pore-UA [Genbank: CAA90791], Sasa-LBA [Genbank: DY733800 and GO062643], Sasa-UBA*0301 [Genbank: AAN75116], Sasa-UBA*1001 [Genbank: AAN75118 ], [Genbank: ABQ59666], Sasa-ZAA*0101 [Genbank: DQ099914], Teni-UA [Genbank: CR724171], Teni-ZE [Genbank: CAF90807], HLA-A2 [Genbank: AAA76608]. Sasa-UDA, Sasa-UGA, Sasa-UHA1, Sasa-UHA2, Sasa-ULA, Sasa-SAA, Sasa-ZAA*0201, Sasa-ZBA, Sasa-ZCA are described in this paper. Click here for file
