# Supplementary figures and images for: Comprehensive analysis of MHC class I genes from the U-, S-, and Z-lineages in Atlantic salmon
Source: BMC Genomics. 2010 Mar 5;11:154. doi: 10.1186/1471-2164-11-154 (PMC2846915; doi:10.1186/1471-2164-11-154)

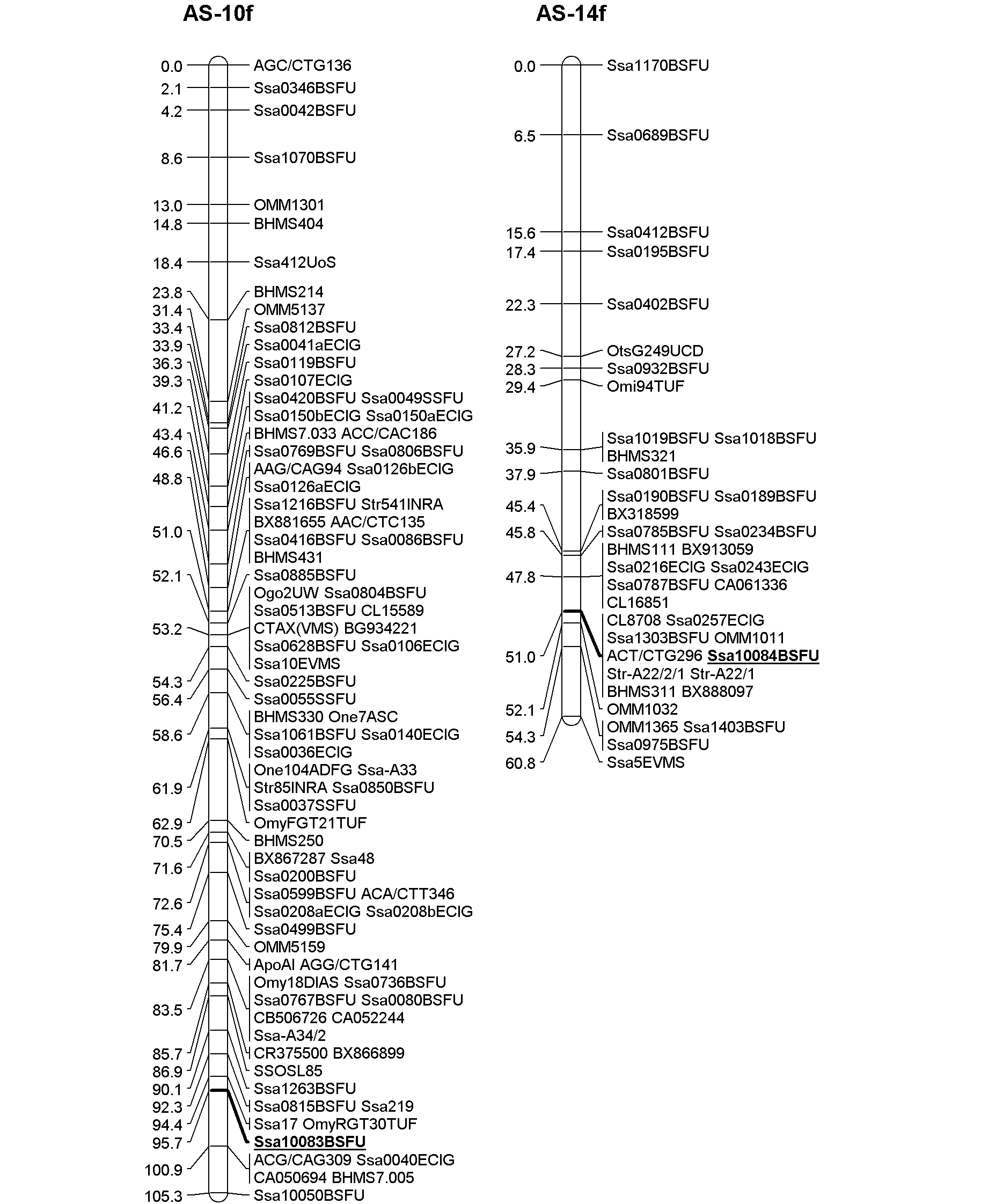

Supplement: Additional file 1 — Linkage groups of SAA and UHA. The positions of SAA (Ssa10083BSFU) and UHA (Ssa10084BSFU) in bold and underlined on linkage group 10 and linkage group 14, respectively of the merged Atlantic salmon female map [17]. [file 1471-2164-11-154-S1.JPEG]
