# Supplementary material for: Comprehensive analysis of MHC class I genes from the U-, S-, and Z-lineages in Atlantic salmon
Source: BMC Genomics. 2010 Mar 5;11:154. doi: 10.1186/1471-2164-11-154 (PMC2846915; doi:10.1186/1471-2164-11-154)
Supplement: Additional file 2 — Phylogeny of teleost MHC class I sequences. Phylogenetic tree analysis by NJ method for full-length amino acid sequences. Consensus trees were based on 1000 bootstrap replications and reported with the bootstrap support values (in percent) indicated above the respective nodes. Sequence references are as follows:Auha-UA [Genbank: AAD37813], Dare-UBA*01 [Genbank: CAA86732], Dare-UAA*01 [Genbank: CAA86731], Dare-UDA*01 [Genbank: AAF20178], Dare-UEA [Genbank: AAH53140], Dare-ZE*0201 [Genbank: CAD12790], Dare-L [Genbank: CAD56801], Furu-UBA [Genbank: AAC41236], Gaac-UAA*01 [Genbank: ABN14358], Gaac-UBA*01, Genbank: ABN14357], Icpu-SAA [Genbank: CK423282], Icpu-UAA [Genbank: AAD08650], Icpu-UBA [Genbank: AAD08648], Icpu-UCA [Genbank: AAD08647], Onmy-SAA [Genbank: AF091779], Onmy-UBA [Genbank: AF287483], Onmy-UCA [Genbank: BAD89552], Onmy-UDA [Genbank: AY523666], Onmy-UEA [Genbank: BAD89553], Onmy-UGA [Genbank: AAP04358 ], Onmy-LAA [Genbank: ABI21842 ], Onmy-LBA [Genbank: ABI21844], Orla-UAA*0101 [Genbank: BAD93265], Orla-UBA*0201 [Genbank: BAB83850], Orla-UCA*0101 [Genbank: BAB63957], Orla-UDA*0201 [Genbank: BAB83843], Orla-UEA*0201 [Genbank: BAB83837], Paol-UA1 [Genbank: BAD13367], Paol-ZE [Genbank: BAD13366], Pore-UA [Genbank: CAA90791], Sasa-LBA [Genbank: DY733800 and GO062643], Sasa-UBA*0301 [Genbank: AAN75116], Sasa-UBA*1001 [Genbank: AAN75118 ], [Genbank: ABQ59666], Sasa-ZAA*0101 [Genbank: DQ099914], Teni-UA [Genbank: CR724171], Teni-ZE [Genbank: CAF90807], HLA-A2 [Genbank: AAA76608]. Sasa-UDA, Sasa-UGA, Sasa-UHA1, Sasa-UHA2, Sasa-ULA, Sasa-SAA, Sasa-ZAA*0201, Sasa-ZBA, Sasa-ZCA are described in this paper. [file 1471-2164-11-154-S2.PPT]

## Slide 1
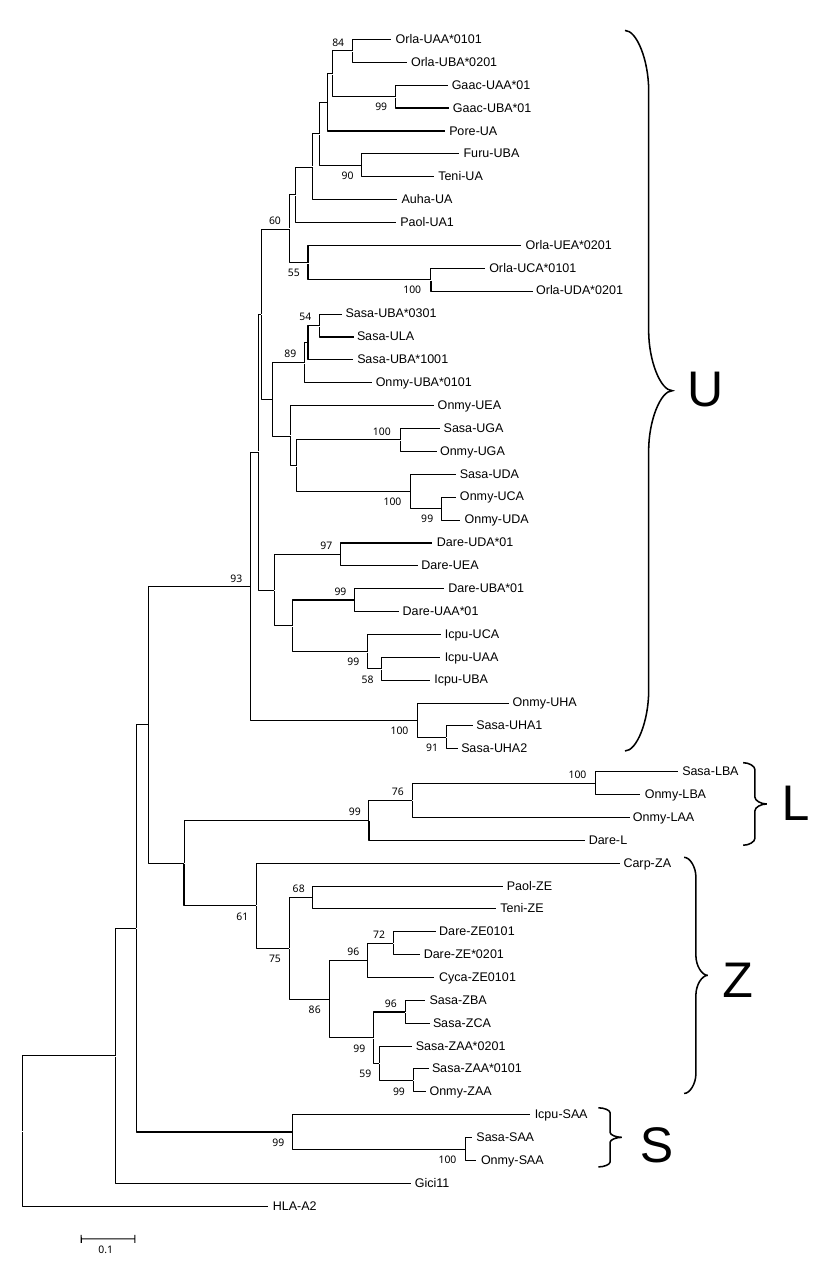

Orla-UAA*0101
84
 Orla-UBA*0201
 Gaac-UAA*01
 Gaac-UBA*01
99
 Pore-UA
 Furu-UBA
 Teni-UA
90
 Auha-UA
 Paol-UA1
60
 Orla-UEA*0201
 Orla-UCA*0101
55
 Orla-UDA*0201
100
 Sasa-UBA*0301
54
 Sasa-ULA
89
 Sasa-UBA*1001
 Onmy-UBA*0101
 Onmy-UEA
 Sasa-UGA
100
 Onmy-UGA
 Sasa-UDA
 Onmy-UCA
100
 Onmy-UDA
99
 Dare-UDA*01
97
 Dare-UEA
 Dare-UBA*01
99
 Dare-UAA*01
 Icpu-UCA
 Icpu-UAA
99
 Icpu-UBA
58
 Onmy-UHA
 Sasa-UHA1
100
 Sasa-UHA2
91
 Sasa-LBA
100
76
 Onmy-LBA
99
 Onmy-LAA
 Dare-L
 Carp-ZA
 Paol-ZE
68
 Teni-ZE
61
 Dare-ZE0101
72
96
 Dare-ZE*0201
75
 Cyca-ZE0101
 Sasa-ZBA
96
86
 Sasa-ZCA
 Sasa-ZAA*0201
99
 Sasa-ZAA*0101
59
 Onmy-ZAA
99
 Icpu-SAA
 Sasa-SAA
99
 Onmy-SAA
100
 Gici11
 HLA-A2
93
0.1
U
L
Z
S
